# Cross-Reactivity of Ragweed Pollen Calcium-Binding Proteins and IgE Sensitization in a Ragweed-Allergic Population from Western Romania

**DOI:** 10.3390/arm92030022

**Published:** 2024-05-30

**Authors:** Lauriana-Eunice Zbîrcea, Maria-Roxana Buzan, Manuela Grijincu, Tudor-Paul Tamaș, Constantina Bianca Vulpe, Ioan Huțu, Virgil Păunescu, Carmen Panaitescu, Kuan-Wei Chen

**Affiliations:** 1 Center of Immuno-Physiology and Biotechnologies, Department of Functional Sciences, Victor Babes University of Medicine and Pharmacy, 300041 Timisoara, Romania; 2 OncoGen Center, Pius Brinzeu County Clinical Emergency Hospital, 300723 Timisoara, Romania; 3 Department of Chemistry-Biology, Institute for Advanced Environmental Research, West University of Timisoara, 300086 Timisoara, Romania; 4 Horia Cernescu Research Unit, Faculty of Veterinary Medicine, University of Life Sciences “King Michael I of Romania”, 300645 Timisoara, Romania

**Keywords:** respiratory allergy, ragweed pollen, calcium-binding proteins, recombinant allergens, Amb a 9, Amb a 10, cross-reactivity

## Abstract

**Highlights:**

**What are the main findings?**

- An IgE reactivity to ragweed pollen calcium-binding proteins (polcalcins) of 25% to rAmb a 9 and 35% to rAmb a 10 was found in a ragweed-allergic population from Romania;
- A strong cross-reactive behavior of rAmb a 9 and rAmb a 10 with rArt v 5 was found, and patients positive to ragweed polcalcins tended to report rather severe respiratory symptoms.

**What are the implications of the main findings?**

- The higher IgE sensitization rate to ragweed polcalcins in western Romania may be due to heavily invaded areas with ragweed as well as due to polcalcin cross-reactivity;
- Recombinant allergens are a useful tool to detect polcalcin sensitization in ragweed-allergic patients, and ragweed polcalcins may be considered for inclusion in the molecular diagnosis.

**Abstract:**

Ragweed pollen allergy is the most common seasonal allergy in western Romania. Prolonged exposure to ragweed pollen may induce sensitization to pan-allergens such as calcium-binding proteins (polcalcins) and progression to more severe symptoms. We aimed to detect IgE sensitization to recombinant Amb a 9 and Amb a 10 in a Romanian population, to assess their potential clinical relevance and cross-reactivity, as well as to investigate the relation with clinical symptoms. rAmb a 9 and rAmb a 10 produced in *Escherichia coli* were used to detect specific IgE in sera from 87 clinically characterized ragweed-allergic patients in ELISA, for basophil activation experiments and rabbit immunization. Rabbit rAmb a 9- and rAmb a 10-specific sera were used to detect possible cross-reactivity with rArt v 5 and reactivity towards ragweed and mugwort pollen extracts. The results showed an IgE reactivity of 25% to rAmb a 9 and 35% to rAmb a 10. rAmb a 10 induced basophil degranulation in three out of four patients tested. Moreover, polcalcin-negative patients reported significantly more skin symptoms, whereas polcalcin-positive patients tended to report more respiratory symptoms. Furthermore, both rabbit antisera showed low reactivity towards extracts and showed high reactivity to rArt v 5, suggesting strong cross-reactivity. Our study indicated that recombinant ragweed polcalcins might be considered for molecular diagnosis.

## 1. Introduction

Ragweed (*Ambrosia artemisiifolia*) pollen is an important respiratory allergen with a wide impact on public health [1,2]. Environmental factors such as climate change and urbanization have led to the increased dispersal of ragweed throughout Europe [3,4], with a constantly increasing prevalence of sensitized individuals in countries like Romania in recent years [5,6,7]. Exposure to ragweed pollen in late summer and fall induces symptoms of allergic rhinitis in sensitized patients and asthma twice as frequently compared to other types of pollen [8,9]. Prolonged exposure to pollen may cause pan-allergen sensitization, which is often related to disease severity [10,11]. Pan-allergens (i.e., profilins and calcium-binding proteins) are usually minor allergens with ubiquitous distribution in all plant families [12]. They are responsible for IgE cross-reactivity with other allergen molecules within the family, due to the high similarity of the protein structure [13,14]. Moreover, the IgE sensitization to calcium-binding proteins from pollen (polcalcins) has been linked with the occurrence of asthma [15]. The molecules Amb a 9 (2-EF hand) and Amb a 10 (4-EF hand) are representative pan-allergens in ragweed pollen and belong to the polcalcin family [16]. The IgE reactivity to Amb a 9 and Amb a 10 has been reported to vary in different European countries, ranging between 10% and almost 30% in some populations [16,17,18]. In Romania, there is a lack of information regarding pan-allergen sensitization in the allergic population, as well as regarding sensitization to polcalcins from ragweed pollen and other common allergen sources. 

Patients’ sensitization to pan-allergens represents a challenge in the diagnosis of allergies due to potential cross-reactivities to homologous proteins, with potential misdiagnosis as a consequence [12,19]. Thus, it is important to investigate the cross-reactivity among polcalcins including Amb a 9 and Amb a 10. In general, cross-reactivity between polcalcins has been intensely studied using representatives from common allergen sources such as birch and timothy grass pollen (i.e., Bet v 4 and Phl p 7) [11,20,21]. Regarding the cross-reactivity of Amb a 9 and Amb a 10, limited cross-reactivity of these two allergens with Phl p 7 and Bet v 4 has been reported [16]. Cross-reactivity of Amb a 9 or Amb a 10 with Art v 5 from mugwort has also been suggested [18], but more data to confirm this is still missing. Besides ragweed, mugwort (*Artemisia vulgaris*) is also an important allergen source in Romania [22] showing similarities with ragweed, and cross-reactivity has already been proven between major and minor allergens of these two weeds [23,24].

The lack of information regarding the cross-reactivity profile and clinical relevance of Amb a 9 and Amb a 10, as well as the IgE sensitization in different populations, highlight the need for more studies on this matter. This can be achieved by the expression of recombinant proteins for diagnostic use, which offer the advantage of higher purity compared to natural allergens [25]. Nowadays, commercial allergen extracts used for diagnosis are still not standardized, and minor allergens such as polcalcins might be underrepresented in these extracts due to degradation [18]. On the other hand, their presence might pose a problem in the extract-based diagnosis for polcalcin-sensitized patients due to cross-reactivity, which could obscure the genuine sensitizing source and give false positive results [26]. Therefore, recombinant allergens provide an alternative for the improvement of allergy diagnosis if clinical relevance is investigated. Also, the use of recombinant allergens may help to test cross-reactivity such as that between Amb a 9, Amb a 10, and Art v 5.

Our study aimed to determine the IgE sensitization rate to recombinant Amb a 9 and Amb a 10 in a population from western Romania, and the potential clinical relevance of these polcalcins, in order to improve the ragweed allergy diagnosis. Furthermore, we aimed to investigate the association between sensitization to ragweed polcalcins and clinical symptoms, as well as potential cross-reactivity between Amb a 9, Amb a 10, and Art v 5 by using rabbit antiserum. These findings may contribute to the current knowledge regarding the importance of ragweed polcalcins in allergy diagnosis.

## 2. Materials and Methods

### 2.1. Patients and Patients’ Sera

Patients (*n* = 87) with a conclusive history of allergy to ragweed pollen, determined by reporting allergic symptoms such as rhinitis and conjunctivitis during ragweed pollen season and by testing positive towards ragweed pollen extract in a skin prick test (SPT) and/or specific IgE from serum, were included in this study. Other sensitizations to common allergen sources were also assessed by SPT or IgE testing using allergen extracts. Informed consent was obtained from all participants in this study before blood collection and sera were stored at −80 °C until use. The study was approved by the local Ethics Committee of Timiș County Emergency Clinical Hospital.

### 2.2. Expression of the Recombinant Polcalcins and Extract Preparation

Three constructs encoding for Amb a 9.0101, Amb a 10.0101, and Art v 5.0101 were designed for *Escherichia coli* expression based on the sequences accessed on the WHO/IUIS Allergen Nomenclature database, www.allergen.org (accessed on 12 October 2022). Each construct, containing a C-terminal hexahistidine tag for purification purposes, was inserted into the pET-27b vector (ATG biosynthetics, Merzhausen, Germany) and transformed into the *E. coli* BL21 Gold (Agilent Technologies, Santa Clara, CA, USA) competent cells by heat-shock. The protein expression was induced by the addition of 0.5 mM isopropyl-thiogalactopyranoside, IPTG (Carl Roth GmbH, Karlsruhe, Germany), and the cells were disrupted by freeze–thaw cycles (3×). The proteins were found in the supernatant and were further dialyzed against Lysis buffer containing 50 mM NaH_2_PO_4_, 300 mM NaCl, and 10 mM imidazole, pH 8, overnight at 4 °C. The proteins were purified by nickel affinity chromatography according to the manufacturer’s recommendation (Qiagen, Hilden, Germany). The purified proteins were further dialyzed in three steps until reaching the final buffer containing 10 mM NaH_2_PO_4_, pH 8.5, and stored at −20 °C until use. 

Ragweed (*Ambrosia artemisiifolia*) and mugwort (*Artemisia vulgaris*) aqueous pollen extracts were prepared by mixing 2 g of pollen (Allergon AB, Ängelholm, Sweden) with 20 mL of sterile phosphate-buffered saline (PBS) (Thermo Fischer Scientific, Waltham, MA, USA) for 4 h at room temperature (RT), and centrifuged at 20.000× *g*, for 30 min, at 4 °C. The obtained supernatant was dialyzed in PBS at 4 °C overnight and the extract was stored at −20 °C until use.

### 2.3. IgE Reactivity towards Recombinant Amb a 9 and Amb a 10 in Ragweed-Allergic Patients

ELISA 96-well plates (MaxiSorp, Thermo Fischer Scientific, Waltham, MA, USA) were coated with 5 µg/mL of recombinant (r) Amb a 9 and Amb a 10. The plates were washed two times with PBS + 0.05% (*v*/*v*) Tween 20 and blocked for 2.5 h with PBS + 0.05% (*v*/*v*) Tween 20 + 3% (*w*/*v*) Bovine Serum Albumin (BSA) at RT. Ragweed-allergic patients’ sera (*n* = 87) were diluted 1:5 in PBS 0.05% + (*v*/*v*) Tween 20 + 0.5% (*w*/*v*) BSA and incubated at 4 °C overnight. After washing the plates, the detection of bound IgE antibodies was performed using the 1:2500-diluted goat anti-human IgE horseradish peroxidase (HRP)-labeled antibody (SeraCare, Milford, MA, USA) for 45 min at 37 °C and 45 min at 4 °C. The plates were washed five times with PBST and the color reaction was induced by adding 100 µL/well of staining solution 2,2-azino-bis (3-ethylbenzthiazoline-6-sulfonic acid) diammonium salt, ABTS (Sigma Aldrich, St. Louis, MO, USA), and 3 µL of H_2_O_2_ in 30 mL ddH_2_O. The absorbance was measured at 405 nm (using 490 nm as a reference) on a microplate reader (Tecan Infinite M200 Pro, Männerdorf, Switzerland). The threshold value was defined as mean OD value plus the standard deviation (3 × SD) of five sera from ragweed non-allergic patients. All measurements were performed in duplicates and the results are expressed as mean values. rAmb a 1.01 produced as described [27] was used to assess the IgE reactivity of patients (*n* = 82) to the major allergen in the ragweed pollen, and the OD values were included in Table S1.

### 2.4. Induction of Allergen-Specific IgG Response in Rabbits 

The New Zealand White (NZW) female rabbits (*n* = 2 per allergen) were immunized three times with 200 µg of rAmb a 9 or rAmb a 10 using Freund’s complete adjuvant once, and twice using Freund’s incomplete adjuvant [28]. The second immunization was administered 4 weeks after the first and the third immunization was administered 3 weeks after the second immunization. The pre-immune serum (PIS) was collected at day 0, the immune serum (IS) was collected 10 days after the second immunization, and the last immune serum (final IS) was collected 10 days after the third immunization. This animal study was approved by the Ethics Committee of the University of Agricultural Sciences and Veterinary Medicine “King Michael I of Romania” from Timisoara.

The rAmb a 9- and rAmb a 10-specific IgG titer was determined in ELISA, as previously described. Rabbit sera (PIS, IS, and final IS) were added to the plate in 6 different dilutions (1 × 10^3^–1 × 10^8^) and incubated overnight at 4 °C. Bound IgGs were detected using 100 µL/well of HRP-labeled donkey anti-rabbit IgG (GE Healthcare, Chicago, IL, USA) diluted 1:2000. The color reaction was induced as described in Section 2.3. All measurements were performed in duplicates and displayed as mean values. rAmb a 9-specific serum and rAmb a 10-specific serum from one rabbit were used for further experiments. The IgG induction to the second rabbit was included in Supplementary Figure S1.

### 2.5. Detection of Potential Cross-Reactivity towards Ragweed and Mugwort Polcalcins and Extracts in ELISA

Cross-reactivity was investigated in ELISA, as described before, and the differences are mentioned here in short. The ELISA plates were coated with 50 µg/mL of ragweed or mugwort pollen extract and 5 µg/mL of either rAmb a 9, rAmb a 10, rArt v 5, or BSA. Rabbit rAmb a 9- and rAmb a 10-specific final IS and PIS for control purposes were diluted 1:1000 and added to the plates. Detection and measurements were performed as mentioned above. OD values of the rabbit PIS were subtracted from the IS.

### 2.6. β-Hexosaminidase Release Assay Using Human Rat Basophil Leukemia Cells

Rat basophil leukemia (huRBL) RS-ATL8 cells, transfected to express the human high-affinity IgE receptor on their surface [29], were cultivated and plated, as previously described [30]. The cells were loaded with 1:10-diluted patient serum from five ragweed-allergic patients, two sensitized to rAmb a 9 (OD ≥ 0.4) and four patients sensitized to rAmb a 10 (OD ≥ 0.4), and incubated overnight at 37 °C, 5% CO_2_. After washing the plate with Tyrode’s buffer prepared with Tyrode’s Salts (Sigma-Aldrich, Vienna, Austria) and NaHCO_3_ (1.0 g/L), the cells were stimulated with 6 different concentrations of rAmb a 9 and rAmb a 10 (starting at 0.01 up to 1000 ng/mL). The detection of the β-hexosaminidase release was evaluated by the addition of 4-Muc (Sigma Aldrich, Vienna, Austria). The measurements were performed in triplicates at 360 nm excitation and 465 nm emission using a microplate reader (Varioskan LUX, Thermo Fisher Scientific, Waltham, MA, USA). The results are shown as the percentage of the total β-hexosaminidase release (induced by the addition of 10% Triton to disrupt the cells). All measurements were performed in triplicates and are displayed as the mean ± SD. 

### 2.7. Statistical Analysis

The statistical analysis in this study was performed with SPSS (Version 20.0, SPSS Inc., Chicago, IL, USA). The Shapiro–Wilk test was used to test the OD values obtained in ELISA on each allergen for normal distribution. The Wilcoxon test was used to compare the OD values of paired samples for rAmb a 9 and rAmb a 10. The chi-square test was used to assess symptom associations in the patients positive and negative to polcalcins. *p* < 0.05 levels were considered statistically significant.

## 3. Results

### 3.1. Patients’ Clinical Characterization

This study investigated 87 ragweed-allergic patients, 48 males and 39 females aged between 18 and 64 years, regarding the symptoms reported during the ragweed pollen season, and the sensitization profiles to other allergen sources confirmed by SPT/IgE tests (Table 1 and Table S1). All patients reported symptoms of rhinitis, 99% reported symptoms of conjunctivitis, 67% reported asthma-like symptoms, and 22% reported skin symptoms. Rhinitis included nasal symptoms such as rhinorrhea, obstruction, pruritus, and sneezing; conjunctivitis included symptoms such as ocular pruritus, irritation, and tearing; asthma-like symptoms were referred to as chest constriction, dyspnea, wheezing, and dry cough; skin symptoms comprised rash, pruritus, and irritation of the skin. Based on the clinical characterization of the patients, 34% were considered sensitized only to ragweed pollen, whereas two thirds (66%) were sensitized to other common allergen sources. The allergen sources most prevalent in our study are grass pollen (39%), followed by house dust mites (30%), mugwort pollen (21%), tree pollen (17%), animal dander (11%), and fungi (6%) (Table 1 and Table S1).

### 3.2. Detection of Specific IgE to rAmb a 9 and rAmb a 10

**Figure 1:**
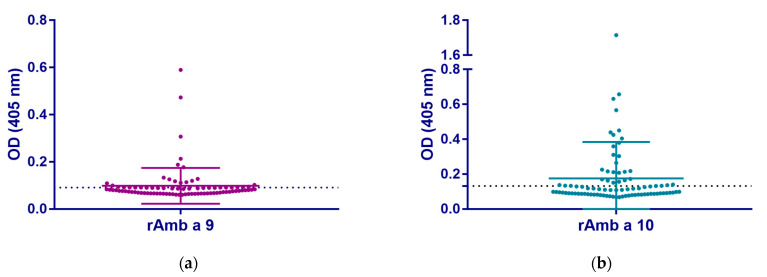
IgE reactivity to ragweed polcalcins (**a**) rAmb a 9 and (**b**) rAmb a 10, as determined in ELISA with sera from patients allergic to ragweed pollen (*n* = 87). The optical density values (OD 405 nm) reflect the levels of IgE antibodies (*y*-axes). The cut-off for rAmb a 9 (0.091) and rAmb a 10 (0.132) is indicated by a dashed line.

The IgE reactivity to ragweed polcalcins, rAmb a 9, and rAmb a 10 was evaluated by ELISA (Table S1) and displayed in Figure 1. The results show that 25.3% of the study population were reactive to rAmb a 9 and 35.6% were reactive to rAmb a 10. The data were not normally distributed in our study population to either rAmb a 9 or rAmb a 10. Statistically significant differences were found between the OD values for rAmb a 9 and rAmb a 10 with higher IgE values for rAmb a 10 (Z = −6.759, *p* < 0.001). Furthermore, all tested patients showed IgE reactivity to rAmb a 1.01 (Table S1).

### 3.3. Allergenic Activity of rAmb a 9 and rAmb a 10 in Basophil Activation Test

**Figure 2:**
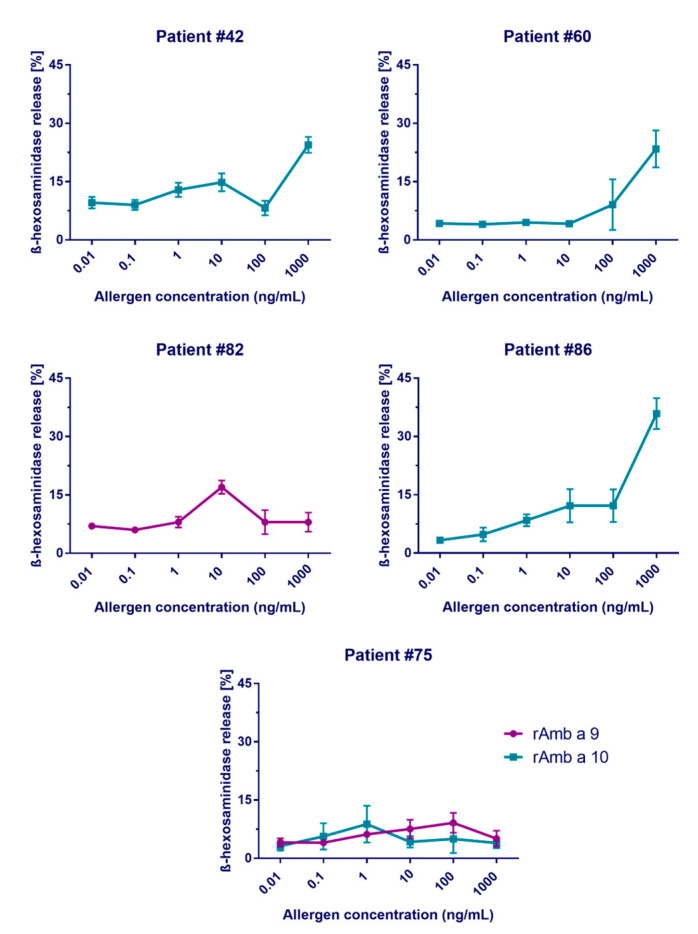
Evaluation of the allergenic activity of ragweed polcalcins in the mediator release assay. Sera from five ragweed-allergic patients were loaded to the huRBL cells and stimulated with different concentrations of rAmb a 9 and rAmb a 10 (*x*-axes). The release of the β-hexosaminidase is expressed as a percentage of the total mediator release +/− SD (*y*-axes.).

The evaluation of the allergenic activity in a mediator release assay showed that rAmb a 10 induced a dose-dependent β-hexosaminidase release in three out of four rAmb a 10-positive patients (patients #42, #60, and #86) with a maximum β-hexosaminidase release between 24–35% at the highest allergen concentration (1000 ng/mL) (Figure 2). rAmb a 9 induced a low β-hexosaminidase release in patient #82, with a maximum β-hexosaminidase release of 16% when cells were stimulated with 10 ng/mL of rAmb a 9, whereas patient #75, sensitized to both allergens, showed almost no degranulation to either rAmb a 9 or rAmb a 10 (Figure 2). 

### 3.4. Association of the Clinical Symptoms with the IgE Response to Ragweed Polcalcins

**Figure 3:**
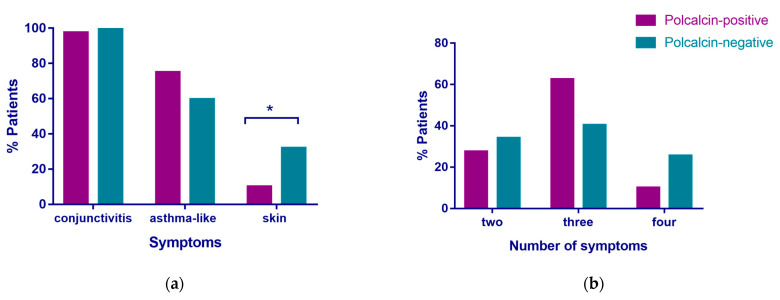
Association of ragweed polcalcin sensitization with clinical symptoms. The relation between (**a**) the prevalence of different allergy symptoms and (**b**) the number of symptoms (*x*-axes), and the reactivity towards either rAmb a 9 or rAmb a 10 is displayed as percentages of patients (*y*-axes). * *p* < 0.05.

We investigated whether there is a difference among the ragweed polcalcin-positive patients (positive to one or both polcalcins, *n* = 40) and polcalcin-negative patients (*n* = 47) regarding the symptom profile. In the polcalcin-positive group, 97.5% of the patients reported symptoms of conjunctivitis, 75% reported asthma-like symptoms, and 10% reported skin symptoms, whereas in the polcalcin-negative group, 100% of patients reported symptoms of conjunctivitis, 59.6% reported asthma-like symptoms, and 31.9% reported skin symptoms (Figure 3a). All patients reported symptoms of rhinitis. Further, the patients were grouped based on the number of the aforementioned allergy symptoms reported during ragweed pollen season (one, two, three, or four). In the polcalcin-positive group, 27.5% of the patients reported two symptoms, 62.5% reported three symptoms, and 10% reported four symptoms, whereas in the polcalcin-negative group, 34% reported two symptoms, 40.4% reported three symptoms, and 25.5% reported four symptoms (Figure 3b). Significant differences were found in the two groups regarding patients reporting skin symptoms (χ^2^ = 4.587, *p* < 0.05) (Figure 3a). No significant differences were found regarding asthma-like symptoms between the two groups, but polcalcin-positive patients reported more asthma-like symptoms (Figure 3a). Also, no statistically significant differences were found in either group regarding the reported number of symptoms. However, more polcalcin-positive patients reported three symptoms.

### 3.5. Detection of rAmb a 9- and rAmb a 10-Specific IgG Antibodies in Rabbit Serum and Reactivity to Weed Pollen Extracts and Polcalcins

**Figure 4:**
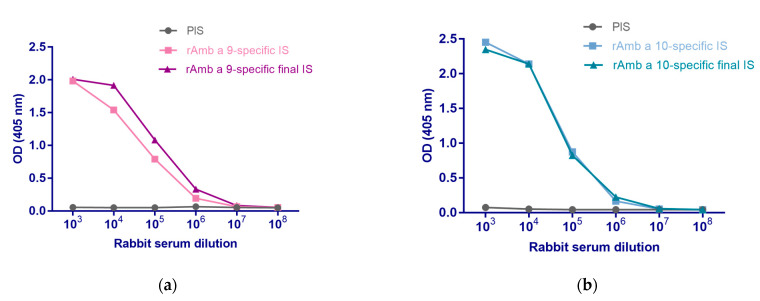
The immune response of rabbits immunized with rAmb a 9 or rAmb a 10. Rabbit pre-immune serum (PIS), immune serum (IS), and final immune serum (final IS) from rabbits immunized with (**a**) rAmb a 9 and (**b**) rAmb a 10 were tested in ELISA against rAmb a 9 and rAmb a 10, respectively. The levels of rAmb a 9- and rAmb a 10-specific IgG antibodies correspond to the optical density values (OD 405 nm, *y*-axes).

The immunization of rabbits with rAmb a 9 and rAmb a 10 induced a high allergen-specific IgG response (Figure 4). rAmb a 9-specific IgG antibodies were detected in rabbit IS and final IS with the highest IgG response at 1:1000 dilution (OD = 2.01), with the signal detected down to 1:1,000,000 dilution (OD = 0.33), contrary to rabbit PIS (Figure 4a). rAmb a 10-specific IgG antibodies were detected in rabbit IS and final IS with similar IgG levels. The highest IgG response was found at 1:1000 dilution (OD = 2.35), with the signal detected down to 1:1,000,000 in rabbit IS and final IS (OD = 0.22), but not in PIS (Figure 4b). A similar IgG induction was found in the second rabbit immunized with rAmb a 9 and in the rabbit immunized with rAmb a 10 (Figure S1).

**Figure 5:**
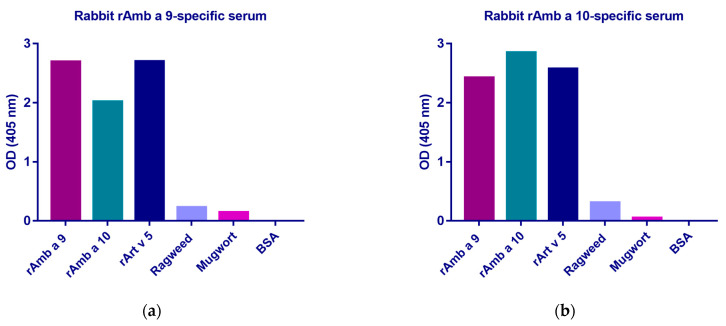
Detection of rAmb a 9- and rAmb a 10-specific IgG antibodies from rabbit allergen-specific serum towards polcalcins and extracts from weeds. rAmb a 9, rAmb a 10, and rArt v 5, as well as ragweed extract and mugwort extract (*x*-axes), were incubated with (**a**) rabbit rAmb a 9-specific serum and (**b**) rabbit rAmb a 10-specific serum. The optical density values (OD 405 nm) correspond to the levels of specific IgG antibodies (*y*-axes).

The rAmb a 9-specific serum reacted with rAmb a 9 and rArt v 5 to the same extent (OD = 2.69) and with rAmb a 10 to a lower extent (OD = 2.02) (Figure 5a). The reactivity of the rAmb a 9-specific serum was low towards ragweed pollen extract (OD = 0.22) and mugwort pollen extract (OD = 0.14), respectively (Figure 5a). The rAmb a 10-specific rabbit serum showed the highest reactivity towards rAmb a 10 (OD = 2.84), followed by rArt v 5 (OD = 2.57) and rAmb a 9 (OD = 2.42). The reactivity of the rAmb a 10-specific serum to ragweed pollen extract was slightly higher (OD = 0.31) than in the case of the rAmb a 9-specific serum, but lower to mugwort pollen extract (OD = 0.05) (Figure 5b). No reactivity was found towards BSA.

## 4. Discussion

Ragweed pollen is a major health problem in Romania, considered the main cause of allergic rhinitis in patients with seasonal allergies, with a highly variable prevalence rate of ragweed sensitization reported in different regions of Romania in the last five years, i.e., 4% in the central region, 18% in the north-western region [31], 24% in the south-eastern region [32], and 48% in southern Romania [33]. Among all the identified ragweed pollen allergens, two belong to the family of polcalcins: Amb a 9 and Amb a 10. The polcalcins are known pan-allergens with ubiquitous distribution in different plants which cross-react with homologous allergens from other sources [12]. However, information regarding the cross-reactivity of Amb a 9 and 10 is still missing, and IgE reactivity to these polcalcins has been reported to vary in different populations [16]. Our study aimed to identify the IgE reactivity to rAmb a 9 and rAmb a 10 in a population of ragweed-allergic patients from western Romania, an area heavily affected by ragweed, to evaluate the potential clinical relevance of these polcalcins and cross-reactivity in order to improve the molecular diagnosis of ragweed allergy.

The evaluation of IgE sensitization towards rAmb a 9 and rAmb a 10 in a ragweed-allergic population from western Romania showed an IgE reactivity of 25% to rAmb a 9 and 35% to rAmb a 10, respectively (Figure 1, Table S1). The high IgE reactivity to rAmb a 1.01 in the tested patients (*n* = 82, Table S1) indicates genuine ragweed sensitization, and none of these patients were monosensitized to ragweed polcalcins. Although our study found a high IgE reactivity to polcalcins rAmb a 9 and rAmb a 10 in the ragweed-allergic population, the patients positive to rAmb a 9 showed a low signal (mean OD value in positive patients = 0.166), whereas in rAmb a 10-positive patients the signal was significantly higher (mean value = 0.322). A similar report investigating the IgE sensitization to rAmb a 9, rAmb a 10, and rArt v 5 found a higher IgE reactivity in an Italian population to these polcalcins (21–28%) compared to an Austrian population (around 10%), but the ELISA signal was weaker in the Italian group [16]. However, differences in the IgE sensitization to polcalcins have been reported before due to geographical locations and different populations [13,34]. The higher IgE reactivity in countries such as Romania and Italy may be due to the heavily infested areas with ragweed. Previous studies found that the areas most affected by ragweed invasion in Europe are the Pannonian Plain, especially Hungary and Romania, the Rhône Valley, and northern Italy [1]. This information may explain the higher IgE reactivity in countries like Romania and Italy compared to Austria. Interestingly, similar findings were observed in the case of the polcalcin Bet v 4, with varying IgE prevalence ranging between 5 and 11% in patients from Northern and Central Europe compared to 27% in Italy [35], suggesting that polcalcin cross-reactivity may also play a role in higher IgE reactivity. 

When testing the allergenic activity, we found that rAmb a 10 induced β-hexosaminidase release in most of the patients tested, with a peak of 35% at the highest concentration of rAmb a 10 (Figure 2), indicating the potency of rAmb a 10 is not that strong and that only high concentrations of rAmb a 10 may induce basophil activation. rAmb a 9 induced low degranulation in two patients tested, indicating the weaker allergenic activity of this allergen. However, these patients were selected based on their higher IgE values to rAmb a 9 and rAmb a 10; thus, when testing patients with lower OD values, we may not obtain similar results, and to prove their clinical relevance, more patients with different reactivities and from different populations need to be tested. Therefore, we further investigated the symptom profiles of patients to see if any differences were to be found in the polcalcin-sensitized patients (Figure 3). Interestingly, we found that patients that tested negative to ragweed polcalcins reported significantly more skin symptoms compared to patients that tested positive to polcalcins, indicating these symptoms were not associated with IgE recognition to ragweed polcalcins and therefore might be associated with other ragweed allergens. Although no statistically significant differences were found regarding asthma-like symptoms, patients sensitized to ragweed polcalcins tended to report more asthma-like symptoms compared to the negative group (75% vs. 59%). Also, more than half (62%) of the polcalcin-sensitized patients tended to report three types of symptoms, i.e., rhinitis, conjunctivitis, and asthma-like symptoms, indicating that polcalcin-positive patients suffer from respiratory symptoms rather than skin symptoms. 

Immunization of NZW rabbits with rAmb a 9 and rAmb a 10 induced a high IgG titer against these allergens. Using the rabbit rAmb a 9- and rAmb a 10-specific serum, we evaluated the potential cross-reactivity between rAmb a 9, rAmb a 10, and rArt v 5. The results demonstrated that the rAmb a 10-specific serum was able to detect rAmb a 9 and rArt v 5, while the rAmb a 9-specific serum was able to react to the same extent towards rAmb a 10 and rArt v 5, indicating strong cross-reactive behavior between these allergens, especially between rAmb a 9 and rArt v 5, which are known to share 80% homology of the sequence [1]. These findings may also explain the higher IgE reactivity to ragweed polcalcins in our area. Exposure to pollen in western Romania is not limited to ragweed but also includes other allergen sources common in this area, such as mugwort, birch, and grasses [22] (Table 1 and Table S1), which are known to contain polcalcins. Hence, patients in our study cohort may be co-sensitized to other polcalcins besides ragweed polcalcins or may cross-react with the latter. Interestingly, an important number of patients in our study were monosensitized to ragweed pollen (34%, Table 1), similar to previous findings in the western region (29–37%) [7,36], while populations from other regions reported less ragweed monosensitization in north-western (27%) and central Romania (20%) [31]. However, more studies in these regions, including a larger number of patients, are needed to confirm these differences.

Furthermore, testing the rabbit rAmb a 9-specific serum and rAmb a 10-specific serum with ragweed and mugwort pollen extracts revealed low reactivity, indicating a low concentration of these polcalcins in the extract (Figure 5). Our findings confirm the drawbacks of using commercial allergen extracts in the diagnosis and treatment of allergies, due to the low concentration or even lack of some allergens in these extracts [26,37]. 

Although the clinical relevance of rAmb a 9 and rAmb a 10 has not been proven and more studies are needed in this direction, we found a strong cross-reactive behavior between ragweed polcalcins and rArt v 5, especially between rAmb a 9 and rArt v 5. These findings are important for our region, where mugwort is also an important allergen source. Moreover, both ragweed and mugwort have the same flowering season (August–September), which makes it difficult to distinguish between genuine ragweed or mugwort allergy when using allergen extracts. Therefore, to improve the accuracy of the allergy diagnosis, we suggest including ragweed pollen calcium-binding proteins in the molecular diagnosis to prevent incorrect diagnosis and treatment recommendations. 

## 5. Conclusions

Our study found a high IgE sensitization rate to rAmb a 9 and rAmb a 10 in a population from western Romania, possibly due to the abundance of ragweed in this region and to polcalcin-related cross-reactivity with other pollens. We observed that patients who tested negative to polcalcins reported more skin symptoms, whereas patients who tested positive to polcalcins tended to report more respiratory symptoms. 

A strong cross-reactive behavior of rAmb a 9 and rAmb a 10 was found towards rArt v 5, and pollen extracts seem to contain low amounts of Amb a 9 and Amb a 10. Therefore, recombinant ragweed polcalcins may be considered for the detection of specific IgE and cross-reactivity in ragweed allergy diagnosis.

## Figures and Tables

**Table 1:** The prevalence of allergic symptoms and other sensitizations of ragweed-allergic patients.

Patients, no.	87
Sex male/female, no.	48/39
Age range (median)	18–64 (34)
Prevalence of symptoms (%)	
Symptoms of rhinitis	100
Symptoms of conjunctivitis	99
Asthma-like symptoms	67
Skin symptoms	22
Sensitized only to ragweed pollen (%)	34
Other sensitizations (%)	
Grass pollen	39
House dust mites	30
Mugwort pollen	21
Tree pollen	17
Animal dander (cat and dog)	11
Fungi	6

## Data Availability

All data are contained in the present article or in the Supplementary Materials.

## Supplementary Materials

The following supporting information can be downloaded at: https://www.mdpi.com/article/10.3390/arm92030022/s1. Figure S1: The IgG response of rabbits immunized with rAmb a 9 and rAmb a 10; Table S1: Characterization of the clinical profiles of patients and the IgE reactivity towards ragweed polcalcins.
