# Supplementary material for: Cross-Reactivity of Ragweed Pollen Calcium-Binding Proteins and IgE Sensitization in a Ragweed-Allergic Population from Western Romania
Source: Adv Respir Med. 2024 May 30;92(3):218–29. doi: 10.3390/arm92030022 (PMC11200559; doi:10.3390/arm92030022)
Supplement: Supplementary file 1 [file arm-92-00022-s001.zip › Supplementary Figure S1.pdf]

Supplementary Figure S1

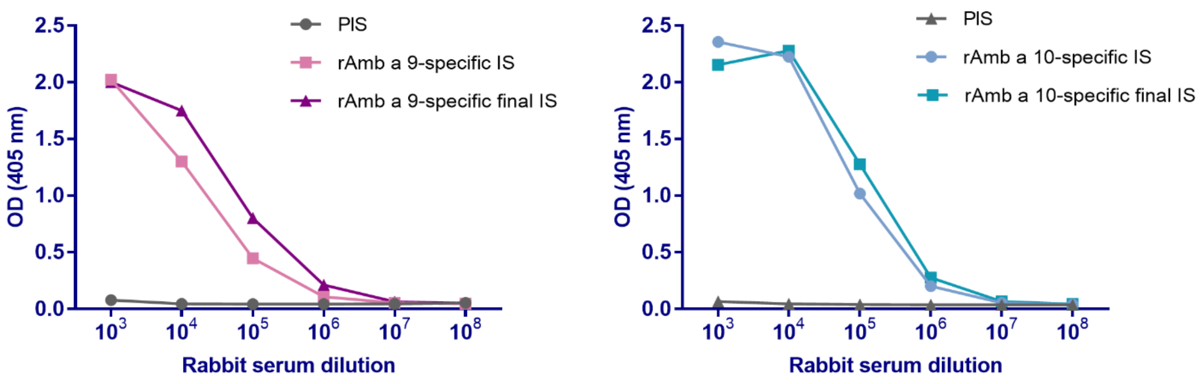

(a) **(b)**

Figure S1. The IgG response of rabbits immunized with rAmb a 9 and rAmb a 10. Pre-immune (PIS), immune serum (IS) and final immune serum (final IS) from rabbit two immunized with (a) rAmb a 9 and (b) rAmb a 10 were tested in ELISA against rAmb a 9 and rAmb a 10, respectively. The optical density values (OD 405 nm) displayed on the y-axes correspond to the levels of IgG antibodies.
